# Magnetic Nanocomposites as Efficient Sorption Materials for Removing Dyes from Aqueous Solutions

**DOI:** 10.1186/s11671-016-1364-2

**Published:** 2016-03-22

**Authors:** Oksana V. Makarchuk, Tetiana A. Dontsova, Ihor M. Astrelin

**Affiliations:** Department of Chemistry, National Technical University of Ukraine “KPI”, Kyiv, 03056 Ukraine

**Keywords:** Magnetic nanocomposite, Magnetite, Saponite, Adsorption, Dyes, Magnetic separation

## Abstract

Magnetic composite sorbents based on saponite clays with different content of magnetite (2–7 wt%.) were synthesized. The samples were analyzed by X-ray diffraction methods, and it was found that the Fe_3_O_4_ in composites is in the nanorange. It has been shown that the magnetic nanocomposites have more developed microporosity and mesoporosity compared to saponite clay. The sorption properties of magnetic nanocomposite sorbents were determined, and the results evidenced that their efficiency is significantly higher than the individual phases of the composite. It was shown that all waste composite magnetic sorbents are successfully removed from the water environment by magnetic separation.

## Background

Dyes and pigments are widely used in the textiles, paper, plastics, leather, food, and cosmetic industries for coloring products. Most of dyes are toxic and must be removed before discharge into receiving streams [1].

To decrease their impact on the environment, a wide range of synthetic dye disposal methods from wastewaters have been developed. The technologies involve adsorption on inorganic or organic matrices, destruction by photocatalysis and/or oxidation processes, microbiological or enzymatic decomposition, etc. [2, 3]. Among various available water treatment technologies, adsorption process is considered more effective because of convenience, ease of operation, and simplicity of design [4]. The most widely used adsorbent for removal of the dyes from aqueous solutions is activated carbon. Various anionic, cationic, and nonionic dyes are removed with high efficiency from aqueous solutions by mesoporous carbon [5]. However, the application of activated carbon for a large-scale wastewater treatment is limited because of its high cost [6].

Cost is a decisive parameter for choice of adsorbent materials. According to [7], a sorbent can be considered low cost if it requires little processing, is abundant in nature, or is a by-product of industry.

In recent years, there has been an increasing interest in clay minerals such as bentonite, kaolinite, diatomite, and saponite for their capacity to adsorb not only inorganic ions but also organic molecules [8, 9]. However, the waste clay particles are removed from the solution after sorption process with considerable difficulties due to their high dispersion. For overcoming this difficulty, clay particles can be magnetized by magnetite and with simple procedure (magnetic separation) removed from water [10–12].

Numerous natural clay adsorbents were used, out of which magnetic composites have gained much attention presently in the removal of dyes from aqueous solution [13]. The high surface area of magnetic composites on clay basis produces rapid adsorption kinetics and thus relatively short contact time [14, 15].

In this article, creating of magnetic nanocomposite sorbents was carried by nanomagnetite modifications of natural saponite clay. Sorption efficiency, structural characteristics, magnetic properties, and magnetic separation process of synthesized samples were investigated.

## Methods

For the study of sorption properties, the magnetite was obtained in the form of magnetic fluid prepared according to the literature [16]. In order to stabilize magnetic fluid, citric acid was used.

Magnetic composite sorbents (MCS) were prepared by impregnation method. The natural saponite clay was sieved to the particle size less than 63 μm. Then 15 g of saponite was dispersed in magnetic fluid. Hence, the synthesis of magnetic composites by impregnation method involves the use of magnetic fluid immediately after its preparation. Therefore, the creation of the MCS 2, MCS 4, MCS 7, and MCS 10 does not demand the use of a stabilizer.

The obtained mixture was mechanically stirred for 0.5 h in order to adsorb magnetite onto saponite. The synthesized sorbent was separated in magnetic filter at magnetic induction of external magnetic field of 66 mT and dried at 60–80 °C for 1 day. The synthesized samples of MCS, saponite, and magnetite are presented in Table 1.

**Table 1:** The sorbents samples

Sample	Content of Fe_3_O_4_, wt%
Saponite	0
MCS 2	2
MCS 4	4
MCS 7	7
MCS 10	10
Magnetite	100

For the study, the process of organic pollutant sorption from aqueous solution dyes of different genesis was used. With the program HyperChem, the 3D models of dye molecules were constructed and geometrically optimized. The greatest linear dimension of the molecule size was adopted by its characteristic size (Table 2).

**Table 2:** Characteristics of organic dyes

Dyestuff	Structural formula	D, nm	М, g/mol
Malachite green		1.331	535.84
Congo red		2.524	696.67
Indigo carmine		1.189	466.36

Powder X-ray diffractions (XRDs) of all sorbent samples were recorded using a Rigaku Ultima IV diffractometer using Cu Kα radiation at 40 KV, 30 mA. Orientated samples were scanned from 2° to 162° 2-theta at 1°/min with a scanning step of 0.0001°/step. The crystallite sizes were calculated using the XRD peak broadening of the peak using Scherer’s formula.

The morphologies of the synthesized products (MCS) were observed using a scanning electron microscope (SEM 106 M).

Structural and adsorption characteristics were measured with the Quantachrome Autosorb (Nova 2200e) by the method of physical adsorption of nitrogen at 77 K. The surface areas were calculated through the Brunauer–Emmett–Teller (BET) equation. The micropore volume *V*_micro_ and the external surface area *S*_*t*_ were calculated from the t-plot method using Harkins-Jura standard isotherm. The value of total pore volume *V*_total_ was estimated from the maximum adsorption at relative pressure close to the saturation pressure. The pore size distribution was obtained from the Barret–Joyner–Halenda (BJH) method.

Adsorption of dyes was carried out in a batch process with 0.5 g of the composites MCS 2, MCS 4, MCS 7, MCS 10, native saponite clay, and magnetic fluid in 50 ml of dye solutions. The obtained suspensions were mechanically stirred for 2 h. After reaching adsorption equilibrium, spent sorbent was separated by centrifugation. The resulting liquid phase was used to determine the residual dye concentration by colorimetric method.

Adsorption properties of sorbents (adsorption capacity *а* (mg/g); efficiency of removal *Х*, (%)) were calculated using Eqs. (1) and (3):

1 $$ x=\frac{\left({C}_0-{C}_{\kappa}\right)\cdot 50}{1000}, $$

2 $$ a=\frac{x}{m}, $$

3 $$ X=\frac{\left({C}_0-{C}_{\kappa}\right)}{C_0}\cdot 100, $$

where *С*_0_ and *С*_к_ are the initial concentration of dye (mg/L) and the residual dye concentration (mg/L), respectively; *a* is the adsorption of *x* (mg) of dye onto 1 g of sorbent (mg/g) from 50 ml of solution.

Magnetic properties of nanocomposites (specific magnetization *σ*_*S*_ (A∙m^2^/kg); magnetic field strength *Н*_с_ (A/m); magnetic induction *B*_*r*_ (mT)) were determined by ballistic magnetometer of Steinberg.

High-gradient magnetic separation of MCS and native saponite clay was studied in magnetic filter. Magnetic induction of external magnetic field was changed from 20 to 200 mT.

The residual concentrations of suspended sorbent particles in an aqueous medium through 5, 60, and 180 min of magnetic separation were determined by turbidimetry method.

## Results and Discussion

**Fig. 1:**
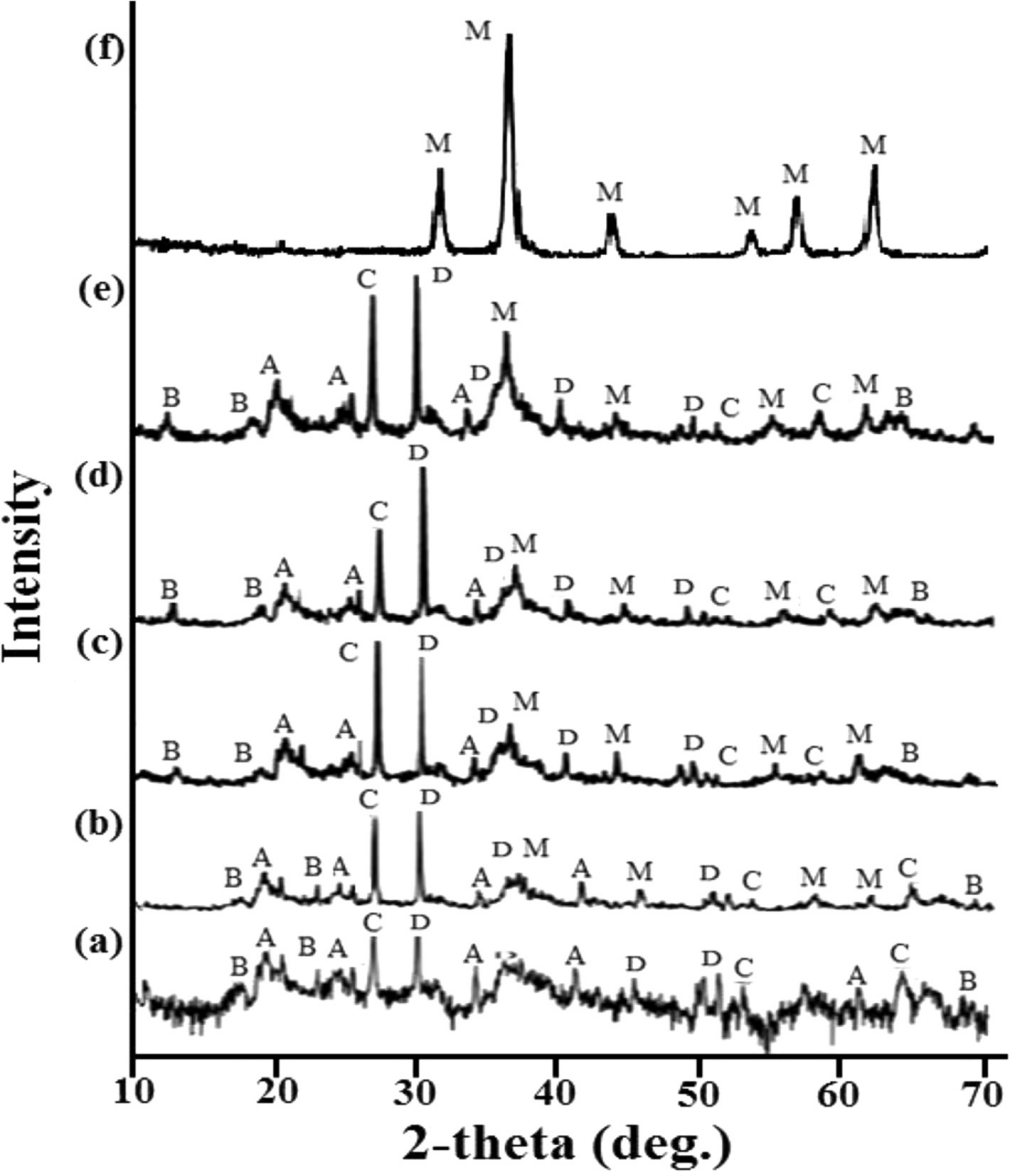
XRD patterns of saponite (*a*), МСS 2 (*b*), МСS 4 (*c*), МСS 7 (*d*), МСS 10 (*e*), and Fe_3_O_4_ (*f*): *A*—saponite NaMg_3_[AISi_3_O_10_](OH)_2_·4H_2_O; *В*—montmorillonite NaMgAlSiO_2_(OH)∙H_2_O; *С*—quartz SiO_2_; *D*—calcite СаСО_3_; and *M*—magnetite Fe_3_O_4_

The XRD patterns of native saponite clay, MCS 2, MCS 4, MCS 7, MCS 10, and Fe_3_O_4_ are shown in Fig. 1. The XRD pattern of the native saponite (Fig. 1a) had indicated peaks that correspond to saponite (00-013-0305), montmorillonite (00-002-0014), quartz (00-001-0649), and calcite (00-002-0623).

Diffraction pattern of magnetite (Fig. 1f) demonstrated strong peaks at 30.72°, 35.38°, 43.72°, 53.64°, 57.24°, and 62.86° 2-theta attributed to the Fe_3_O_4_ (01-071-6336). No other impurities were observed. Hence, the method of Elmore allows to obtain the pure magnetic modifying agent.

The crystal plane diffraction peaks of composite sorbents (Fig. 1b–e) detected the presence of inherent phases of native saponite clay and found peaks that corresponded Fe_3_O_4_. The intensity of the peaks of magnetite was increased with increasing of Fe_3_O_4_ content.

The crystallite sizes and the unit cell parameters of magnetite and magnetite in magnetic composites were calculated and reported in Table 3.

**Table 3:** X-ray analysis of samples MCS 2, MCS 4, MCS 7, MCS 10, and Fe_3_O_4_

Sample	Average size of crystallites of Fe_3_O_4_, nm	Cell parameters, nm		
		а	b	c
MCS 2	2.4	0.847	0.847	0.847
MCS 4	6.2	0.833	0.833	0.833
MCS 7	9.6	0.833	0.833	0.833
МКС3 10	7.4	0.838	0.838	0.838
Fe_3_O_4_	17.9	0.835	0.835	0.835

As stated in Table 2, magnetite in composite sorbents was obtained in the nanorange. The average crystallite size of Fe_3_O_4_ nanoparticles were ranged from 2 to 10 nm and crossed through a maximum for MCS 7.

The calculated pure magnetite crystalline size was exceeded Fe_3_O_4_ crystallite size in nanocomposite sorbents in three times due to aggregation of nanoparticles of magnetite in the magnetic liquid over time. So, the saponite clay matrix had stabilized the nanoparticles of Fe_3_O_4_.

**Fig. 2:**
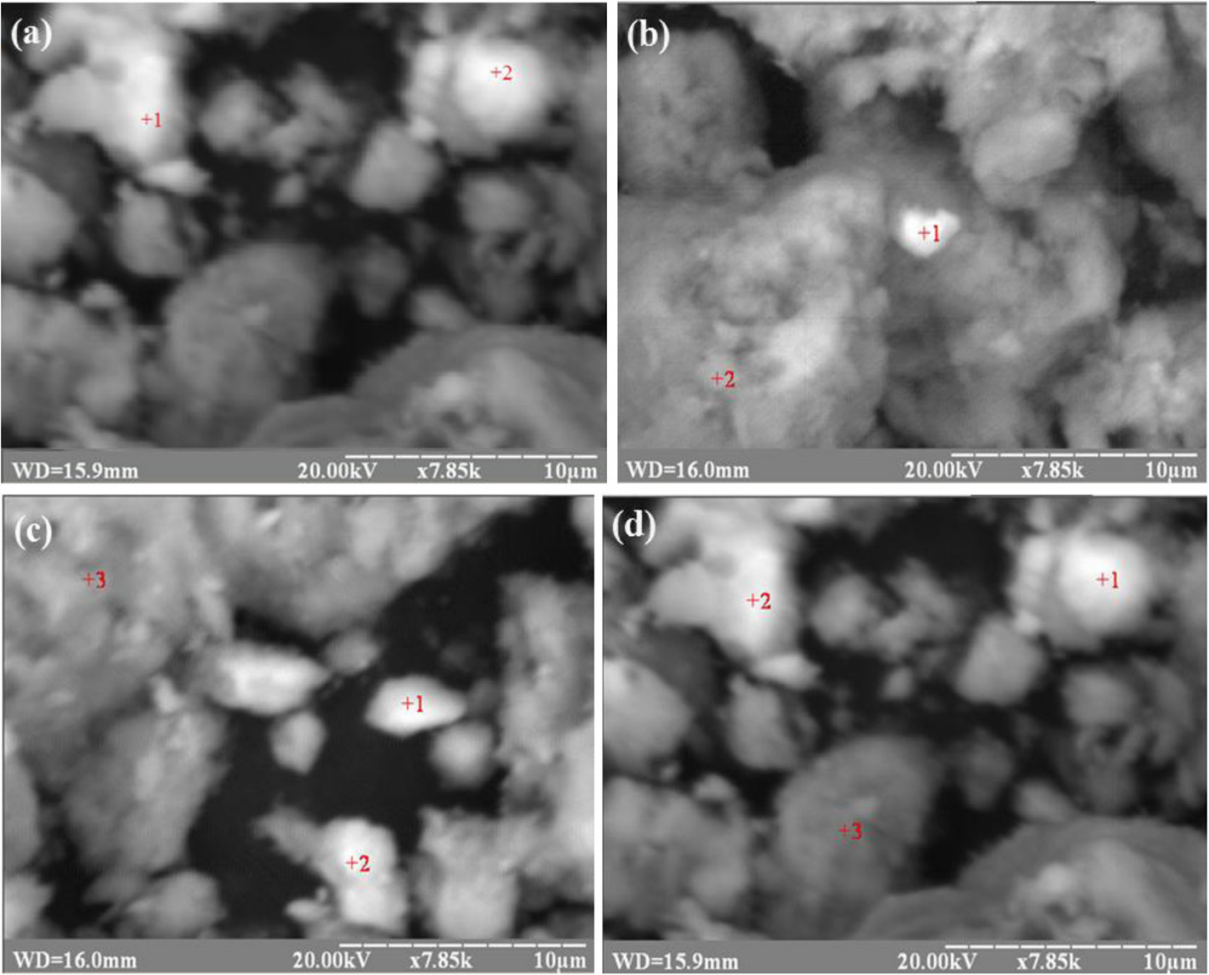
SEM images of surface of the samples MCS 2 (**a**), MCS 4 (**b**), MCS 7 (**c**), and MCS 10 (**d**)

Figure 2 exposes the SEM micrographs of all nanocomposite sorbents. These images show that magnetite had precipitated on the surface of saponite unequally. Table 3 presents the selective sorbent surface chemical analysis of the nanosorbent MCS. As seen from Table 4, Fe content on the surface of saponite matrix is increased with increasing amounts of magnetite.

**Table 4:** The selective sorbents surface chemical analysis of the nanocomposites MCS

Chemical element	Contents, wt%			
Point	1	2	1	2
Sample	MCS 2		MCS 4	
Mg	1.1	0.4	1.8	5.7
Al	3.1	0.6	1.9	12.8
Si	14.3	2.6	6.2	36.8
Ca	6.6	1.7	1.7	3.4
Fe	74.9	94.7	88.4	41.3
Sample	MCS 7		MCS 10	
Mg	0.4	2.7	1.1	0.4
Al	0.7	3.0	1.6	0.7
Si	2.5	9.8	6.2	2.2
Ca	4.2	7.6	0.8	0.3
Fe	92.2	76.9	90.3	96.4

Table 5 summarizes the characteristics of porous structure of nanocomposite sorbents and saponite. The specific surface area of sorbents is increased with increasing amounts of magnetite. For the samples MCS 2, MCS 4, and MCS 7, it is explained by development of micropore and mesopore structure and formation of nanosized magnetite layer on the surface of the saponite pores. Preferred pore diameter of the samples MCS 2, MCS 4, and MCS 7 is in the range of 4 to 4.5 nm.

**Table 5:** Characteristics of nanocomposite sorbents and saponite porous structure

Characteristic	Saponite	MCS 2	MCS 4	MCS 7	MCS 10
Specific surface area *S*, m^2^/g	34.64	53.03	55.76	53.38	69.07
Surface area of micropores *S*_micro_, m^2^/g	9.59	17.38	18.10	12.16	–
External surface area of pores *S*_ext_, m^2^/g	25.05	35.64	37.66	41.68	69.07
Total pore volume *V*_total_, ml/g	0.1139	0.1441	0.1455	0.1464	0.3058
Volume of micropores *V*_micro_, ml/g (%)	0.0054 (4.74)	0.0089 (6.18)	0.0091 (6.25)	0.0064 (4.37)	–
Volume of mesopore *V*_meso_, ml/g (%)	0.0307 (26.95)	0.1109 (76.96)	0.1137 (78.15)	0.1210 (82.65)	0.2876 (94.05)
Average pore diameter *d*_a_, nm	12.00	10.39	10.0	10.56	17.66
Preferred pore diameter *d*_p_, nm	60.57	4.32	4.21	4.27	18.52

For the sample MCS 10, blocking of micropores by Fe_3_O_4_ nanoparticles is observed. Also, in this sample, an intensive formation of mesopores with comparatively large preferred diameter of 18.5 nm was detected. Investigation of magnetite porous structure was not carried out since from the literature [17, 18] was known that Fe_3_O_4_ is nonporous sorbent.

Table 6 provides the information about the adsorption capacity and the efficiency of organic dye removal by saponite, magnetic liquid, and nanocomposite sorbents on their basis. The MCS 2, MCS 4, and MCS 7 were the most effective sorbents. Namely, adsorption capacity of MCS in respect of all dyes is increased with increasing magnetite content from 2 to 7 wt%. However, saponite modification by magnetite in an amount of 10 wt% caused the deterioration of MCS 10 sorption properties. These obtained data are agreed with the characteristic of nanocomposites porous structure.

**Table 6:** Adsorption values of organic dyes adsorption on MCS, Fe_3_O_4_, and saponite clay

Sorbent	Malachite green		Congo red		Indigo carmine	
	*а*_*t*_, mg/g	*Х*, %	*а*_*t*_, mg/g	*Х*, %	*а*_*t*_, mg/g	*Х*, %
Saponite	105.7	26.4	30.7	10.2	62.1	20.7
MCS 2	159.1	39.8	73.0	24.3	110.3	36.8
MCS 4	283.2	70.8	126.9	42.3	124.1	41.4
MCS 7	324.5	81.1	176.9	59.0	148.3	49.4
MCS 10	86.8	21.7	46.1	15.4	51.7	17.2
Fe_3_O_4_	36.7	9.2	59.6	19.9	44.8	14.9

The efficiency of removal of Malachite green, Congo red, and Indigo carmine, for example, by MCS 7 was in 3, 6, and 2.5, accordingly, times higher than by saponite clay. Sorption capacity of  MCS 7 relatively Malachite green, Congo red, and Indigo carmine is exceeded sorption capacity value of magnetite at 9, 3, and 3.5 times, respectively (Table 6). Thus, for the created magnetic nanocomposite sorbents, synergistic effect is observed.

The specific saturation magnetization, magnetic field strength, and magnetic induction of synthesized MCS samples and Fe_3_O_4_ are reported in Table 7. According to previous studies [19], saponite is paramagnetic; therefore, its magnetic characteristics were not considered.

**Table 7:** Magnetic characteristics of samples MCS

Sample	*σ*_*S*(10)_, А∙m^2^/kg	*Н*_*с*_, A/m	*B*_*r*_, mT
MCS 2	2.2	–	–
MCS 4	3.0	–	–
MCS 7	4.5	954.9	1.2
MCS 10	6.5	954.9	1.1
Fe_3_O_4_	90.0	501.3	3.5

As is known [20], the magnetite change of reversal mechanism from reorientation of magnetic moments (single-domain state) to displacement of domain walls (poly-domain state) occurred at about 30 nm. In our case, the Fe_3_O_4_ crystallites in magnetic nanocomposites were formed almost identical size (2–10 nm); therefore, the specific saturation magnetization of MCS samples was approximately the same. Hence, the nanoscale particles of magnetic material were formed with the same magnetization and arrangement of spins in one direction.

Also, from Table 6, we can clearly see that the magnetic field strength and magnetic induction of MCS 2 and MCS 4 were disappeared. This can be explained as follows. After the transfer of magnetic oxide nanoparticles to single-domain state, there are some critical values of their size [20], at which they are in super paramagnetic state. In our case, apparently, the critical diameter of Fe_3_O_4_ was 5–6 nm. Magnetic properties of composite magnetic sorbents are agreed with the results of X-ray analysis (Table 3).

The resulting magnetic characteristics of composites MCS were also confirmed with the values of residual turbidity of the water environment after magnetic separation (Table 8). Magnetic materials with higher values of coercive force *Н*_с_ (MCS 7 and MCS 10) were removed in an external magnetic field with greater efficiency.

**Table 8:** Characteristics of the magnetic separation process of samples MCS

Sample	*τ*, min	*C*, mg/dm^3^
Saponite	5	419
	60	241
	180	154
MCS 2	5	252
	60	58
	180	9
MCS 4	5	97
	60	32
	180	7
MCS 7	5	52
	60	18
	180	<0.5
MCS 10	5	38
	60	7
	180	<0.5

The magnetic separation process of magnetite was not explored because Fe_3_O_4_ is ferromagnetic, and it is separated immediately in the magnetic field with the induction of 20 mT.

Consequently, obtained magnetic composites MCS 2, MCS 4, and MCS 7 have greater sorption properties with respect to dyes of different genesis in comparison with saponite clays and magnetite. Magnetic nanocomposites can be effectively removed from the aqueous solution by magnetic separation. Sludge of magnetic spent sorbent is proposed to utilize in the production of ceramic bricks. However, previous data showed that creation of MCS 2 is the most appropriate because the cost of it is at least two times less than the rest of the sample composites.

## Conclusions

Magnetic nanocomposite sorbents based on cheap saponite clay and nanosized magnetite were obtained by the easy way. It was established that the introduction of magnetite in an amount of 2–7 wt%, the specific surface area of the composites was increased in two times compared to the saponite. Thus, the microporous and mesoporous structure was developed by the formation of nanosized Fe_3_O_4_ layer on the surface of the saponite clay pores.

The obtained nanocomposites have greater sorption properties with respect to dyes of different genesis in comparison with saponite clays and magnetite. Hence, the synergy effect was found. In our view, this was caused by the stabilization of magnetite nanoparticles on the surface saponite and by the formation of micropore and mesopore structure.

The research results of sorbent magnetic characteristics have established that magnetite particles in the composition of nanocomposites are a single-domain. It was shown that magnetic nanocomposite sorbents were easily and effectively removed from the water environment by magnetic separation.
